# Suicide prevention in Japanese cancer care hospitals: a topic modeling analysis of manuals and workflows

**DOI:** 10.3389/fpsyt.2026.1743690

**Published:** 2026-01-21

**Authors:** Fumiko Kumada, Ken Kurisu, Masako Okamura, Tatsuo Akechi, Yumi Matsumura, Yosuke Uchitomi, Maiko Fujimori

**Affiliations:** 1 Division of Survivorship Research, National Cancer Center Institute for Cancer Control, Tokyo, Japan; 2 Department of Cancer Survivorship and Digital Medicine, The Jikei University School of Medicine, Tokyo, Japan; 3 Department of Psychiatry and Cognitive-Behavioral Medicine, Nagoya City University Graduate School of Medical Sciences, Nagoya, Japan; 4 Department of Patient Safety, Kyoto University Hospital, Kyoto, Japan

**Keywords:** cancer, health services administration, hospitals, neoplasms, suicide prevention, topic modeling

## Abstract

**Introduction:**

People with cancer in Japan have an elevated risk of suicide, yet little information is available regarding in-hospital suicide prevention manuals and workflows. This study investigated the strategies and frameworks described in manuals and workflows developed by Designated Cancer Care Hospitals.

**Methods:**

Using snowball sampling, suicide prevention manuals and workflows for patients with cancer were collected from 14 Designated Cancer Care Hospitals. The textual data were quantitatively analyzed using topic modeling, specifically latent Dirichlet allocation, a text-mining technique. Topics were extracted and labeled through discussions with suicide prevention experts. Based on these topics, we proposed a set of recommended components for suicide prevention manuals and workflows for people with cancer.

**Results:**

In total, 329 paragraphs from the manuals and workflows of 13 hospitals were analyzed, yielding 20 topics. Among these, six topics were commonly addressed across hospitals, covering prevention (e.g., “Types of Suicide Hotspots and Examples of Countermeasures”), intervention (e.g., “Procedures and Collaboration System for Managing Patients with Suicidal Ideation”), and postvention (e.g., “On-Site Response Procedures Upon Discovering a Suicide”). In addition, six topics were described in greater length by certain hospitals, reflecting their unique institutional characteristics (e.g., “Overview of Suicide Prevention Measures”).

**Conclusions:**

Commonly shared topics should be prioritized in suicide prevention manuals and workflows for patients with cancer. Conversely, hospital-specific topics may serve as useful references tailored to the distinct characteristics of each hospital. Factors such as hospital size, geographic location, and clinical practice should be considered when determining the content of these manuals and workflows.

## 1 Introduction

People with cancer have a substantially higher risk of suicide than the general population. A systematic review of 62 studies involving approximately 47 million individuals found a significantly elevated suicide risk among patients with cancer (1). In Japan, analyses based on the National Cancer Registry demonstrated that suicide risk is particularly high immediately after diagnosis, reaching approximately 4.4 times that of the general population within the first month (2, 3). Despite widespread knowledge of this risk, a systematic review of intervention studies examining suicide, suicide attempts, self-harm, and suicidal ideation as outcomes found no interventions that clearly demonstrated a preventive effect on suicide among individuals with cancer (4).

In response to this gap between risk recognition and effective prevention, suicide prevention has become an explicit policy priority in Japanese cancer care. The 2022 revision of the Guidelines for the Development of Designated Cancer Care Hospitals (DCCHs) requires hospitals to establish standardized in-hospital workflows to address suicide risk among people with cancer, including clearly defined response procedures and coordination with relevant organizations (5). This focus was reinforced in the Fourth Basic Plan to Promote Cancer Control Programs announced in 2023, which identified suicide among people with cancer as a critical issue (6). In addition, an analysis of patient safety reports from 620 hospitals underscored the need to strengthen awareness and implement suicide prevention measures within medical institutions (7). However, a recent nationwide survey revealed that approximately one quarter of DCCHs still lacked implemented suicide prevention manuals or workflows as of February 2023 (8).

Various organizations, including those in Japan, have published online resources, manuals, and guidelines on suicide prevention (9–14). Additionally, the National Cancer Center (NCC) has published a guidance document specifically addressing suicide prevention in cancer care (hereafter referred to as the “NCC Guidelines”) (15). These materials outline the epidemiological characteristics of suicide among individuals with cancer and recommend key components for effective prevention. Based on these resources, DCCHs are expected to develop their own action-oriented manuals and workflows tailored to their specific contexts. However, no previous research has examined the actual content of the suicide prevention manuals currently used in medical hospitals, which may provide insights into how suicide prevention is operationalized internationally, the extent to which practices align with national and international guidelines, the presence of locally developed strategies, and the practical challenges involved in implementation. Furthermore, comparing manuals across multiple institutions may help identify best practices and inform future efforts toward standardization and quality improvement of suicide prevention manuals.

Therefore, we aimed to examine the approaches and organizational frameworks for suicide prevention strategies in DCCHs by collecting suicide prevention manuals and workflows and conducting quantitative content analysis using text mining techniques.

## 2 Materials and methods

### 2.1 Data sources

A researcher with expertise in suicide among people with cancer contacted DCCHs and used snowball sampling to collect internal documents—such as manuals and workflows—related to suicide prevention and postvention for people with cancer between April and May 2023. Documents were obtained through direct requests to the hospitals, with the understanding that institutional identities would remain confidential. The first hospital contacted was the institution affiliated with a member of the research project team (Y.M.), who has expertise in suicide prevention among people with cancer. This hospital served as the starting point for snowball sampling and was asked to recommend other DCCHs that had implemented relevant suicide prevention manuals or workflows. Recruitment continued until the authors considered that newly obtained documents did not yield substantively new content.

Ethical approval was not required for this study because no human participants were involved.

### 2.2 Analysis

To extract the key components described in the collected suicide prevention manuals and workflows, we employed topic modeling, a text mining technique for identifying latent thematic structures in documents. Specifically, we conducted unsupervised machine learning analysis using latent Dirichlet allocation (LDA) (16), which identifies latent topics by analyzing word co-occurrence patterns across a document corpus (17).

Topic extraction was conducted at the paragraph level rather than at the sentence level because a sentence-based analysis would yield overly fine-grained topics and complicated interpretations. Figures and tables in the documents were converted into complete sentences by using the original words and phrases and supplementing them with grammatical elements, such as conjunctions and auxiliary verbs, so that the content formed coherent statements. Each converted figure or table was then treated as a single paragraph in the analysis. Furthermore, of the eight major classes of independent words in Japanese (nouns, verbs, adjectives, adjectival verbs, adverbs, determiners, conjunctions, and interjections), only nouns and verbs were included in the analysis, as the remaining classes are primarily modifiers or connectors, rather than core semantic content. Accordingly, morphological analysis was used to extract only nouns and verbs for topic modeling. Topic analysis using LDA requires a predefined number of topics. We determined the optimal number of topics based on the perplexity, a metric that reflects the predictive performance (18).

The names of the topics extracted through LDA were determined via a three-round consensus process among four researchers with expertise in suicide prevention among people with cancer. First, paragraphs associated with each topic were classified into more detailed subgroups, and a provisional name was assigned to each subgroup. Subsequently, a name was proposed for each topic based on common themes identified across the subgroups. Each researcher suggested their own subgroups and topic names, and final decisions were made through discussion. Furthermore, based on the extracted topics and with reference to the nine recommended components of suicide prevention outlined in the general section of the NCC Guidelines (15), the researchers reached a consensus on a set of recommended components for suicide prevention manuals and workflows for people with cancer.

All analyses in this study were performed using KH Coder 3 (19).

## 3 Results

### 3.1 Document characteristics

We contacted 23 hospitals and collected suicide prevention manuals and workflows from 14 of them, including general (n = 5), university (n = 5), and cancer specialty (n = 4) hospitals (**Table 1**). The remaining hospitals (n = 9) declined to provide documents for reasons including ongoing material development or the absence of any relevant documentation in use. The manual from Hospital H was cited in the references of the manual from Hospital K (**Table 1**), and the two were similar regarding structural composition and textual phrasing. Therefore, the manual from Hospital K was excluded from the analysis, and the final analysis was conducted using the manuals and workflows from 13 hospitals.

**Table 1:** Summary of collected manuals and workflows from 14 hospitals.

Hospital ID.	Type of DCCH	Type of hospital	Format of manual		Pages	Paragraphs	Japanese characters
			Standalone	Partial			
A†	Prefectural	University	●		28	85	20,523
B	Prefectural	General	●		6	17	4,086
C†	Prefectural	Cancer		●	4	8	2,869
D	Prefectural	Cancer		●	3	8	1,888
E	Prefectural	Cancer		●	2	5	1,060
F	Prefectural	Cancer		●	2	3	885
G†	Regional	University		●	18	73	14,247
H†	Regional	University	●		16	52	9,242
I†	Regional	University	●		2	8	1,485
J†	Regional	University	●		1	1	489
K†	Regional	General	●		15	40	8,645
L†	Regional	General		●	10	28	5,273
M†	Regional	General	●		10	28	7,163
N†	Regional	General	●		6	13	1,353

DCCH stands for Designated Cancer Care Hospital.

Regarding the format of manuals, “standalone” refers to hospitals that operate with an independent document, whereas “partial” indicates those incorporating content as part of another manual (e.g., a general safety manual).

In the Type of hospital column, “University,” “General,” and “Cancer” refer to university hospitals, general hospitals, and cancer centers, respectively.

Cells marked with † denote hospitals whose manuals explicitly mention “cancer.”

Character and paragraph counts included only the main text of the manuals, excluding the tables of contents and reference lists.

The documents varied in length, ranging from a concise one-page suicide prevention workflow (approximately 500 Japanese characters, equivalent to 200 English words) to a comprehensive 28-page report comprising 85 paragraphs and over 20,000 Japanese characters (approximately 8,000 English words). On average, the documents were 8.8 pages in length, containing 26.4 paragraphs and approximately 5,400 characters. Some hospitals used the suicide prevention manual as a standalone document, whereas others incorporated it into a broader manual, such as a general medical safety manual. Among these, 10 hospitals (**Table 1**) explicitly used the word “cancer” and included detailed descriptions of topics, such as the current state of suicide risk among people with cancer, risk factors, and responses to patients with advanced-stage cancer.

Manuals from 13 hospitals were segmented into 329 paragraphs. Morphological analysis yielded 41,183 words, of which 3,180 were unique. Among these, 5,540 words (2,429 unique) were nouns or verbs used in the analysis.

### 3.2 Topic modeling results

Based on perplexity, the number of topics was set to 20. The occurrence probabilities of these topics were calculated for each of the 329 paragraphs (the sum of these probabilities was equal to one for each paragraph). The relationship between topic-occurrence probabilities and the estimated number of paragraphs exceeding each probability threshold is presented in **Supplementary Figure 1**. Considering this trade-off between interpretability and coverage, a threshold of 0.15 was adopted, resulting in the selection of 153 paragraphs (47% of the total) for subsequent analyses. When this threshold was applied, 10 of the 153 paragraphs were assigned to more than one topic (**Supplementary Figure 1**).

To assess the impact of topic-occurrence probabilities in relation to manual lengths, we calculated the proportion of paragraphs exceeding the probability threshold relative to the total number of paragraphs (**Supplementary Figure 2**). The correlation coefficient between the total number of paragraphs and this proportion was −0.37, indicating a negative association. This suggests that manuals containing a larger number of paragraphs did not disproportionately influence topic identification, nor did manuals with fewer paragraphs systematically show weaker contributions to the topic modeling results.

### 3.3 Thematic content analysis

The subgroups within each topic were classified into one of three categories of suicide prevention: prevention (measures taken prior to crisis intervention during routine care), intervention (measures during crisis intervention), and postvention (measures implemented after a suicide-related incident). Two additional categories were added for topics that did not align with any of the three phases:

- Overview, which included epidemiological information on suicide among people with cancer and the broader public health and societal importance of suicide prevention.
- System, which included descriptions of interdepartmental and inter-organizational coordination structures, as well as routine communication systems.

The subgroup names and their classifications are presented in **Table 2**.

**Table 2:** List of 20 topics and subgroup names.

Topic No.	Topic title	Subgroup	Category					Type	
			Over view	Type of prevention			Sys tem	C	S
				Pre ven tion	Inter ven tion	Post ven tion			
1	Suicide Risk in People with Cancer	Epidemiology of Suicide Risk in People with Cancer (4)†	●						
		Suicide Risk Strategies in Designated Cancer Care Hospitals (1)†	●						
		Risk Factors for Suicide in People with Cancer (1)†	●						
2	Overview of Suicide Prevention Measures	Prevention, Intervention, and Postvention Strategies for Suicide (4)†	●						•
		Suicide Prevention as a Social Responsibility (2)	●						
		Practical Examples of Suicide Prevention: Counseling, Education, Support, and Systems (2)	●						
		Suicide Postvention (1)	●						
		References for Suicide Prevention Manuals (1)	●						
3	Types of Suicide Hotspots and Examples of Countermeasures	Examples of Hotspot Types and Preventive Measures (10)		●				•	
4	Explanation of Total Pain	Total (Holistic) Pain (1)		●					
5	Suicide Risk Assessment and Response	Warning Signs that Elevate Suicide Risk and Corresponding Responses (5)†		●	●				
		Documentation of Risk Assessment (1)		●	●				
6	Risk Factors for Suicide and Assessment Methods	Suicide Risk Assessment Checklist (Source: Journal of Patient Safety Promotion, Issue 17) (4)		●				•	
		Risk Factors for Suicide (7)†		●					
		Patient Checklist for Suicide Risk Assessment (1)		●					
		Factors Contributing to Inpatient Suicide (1)		●					
		Checklist for Patients with Suicidal Ideation (1)			●				
7	Procedures and Collaboration System for Managing Patients with Suicidal Ideation	Procedures and Coordination for Providing Care for Patients with Suicidal Ideation (7)†			●			•	
		Coordination and Procedures for Sharing Mental Health Information Between Outpatient and Inpatient Units (1)		●	●				
		Response and Coordination Procedures for Inpatients After Suicide Attempts (1)			●				
8	Screening Methods for Emotional Distress	Questionnaire on Ease of Daily Living (5)†		●					•
		Screening Using the Integrated Palliative Care Outcome Scale (1)†		●					
		Post-Incident Staff Support (1)				●			
9	Examples of Responses to Suicide-Related Behaviors	Methods for Responding to Patients with Suicidal Ideation (4)		●	●				•
		Methods for Responding to Patients After Suicide Attempts (1)			●	●			
		Methods for Communicating with Other Patients After a Suicide (1)				●			
		Medical Provider-Related Factors in Suicide: Communication “Don’ts” (1)†		●	●				
10	Measures to Address Issues Faced by Patients and Bereaved Families	Responding to Patient Issues as Suicide Risk Factors (3)		●	●				
		Support for Problems Faced by Bereaved Families (3)				●			
11	Examples of Communication Methods with Patients, Bereaved Families, and Other Patients	Communication Methods During Crisis Intervention (12)			●			•	
		Communication with Patients Using the Questionnaire on Ease of Daily Living (1)†		●	●				
		Communication with Other Patients After a Suicide (2)				●			
		Communication with Bereaved Families After a Suicide (1)				●			
12	Psychological Reactions, Including Grief, and Related Support	Psychological Reactions Including Grief Among the Bereaved and Corresponding Support (8)				●			•
		Grief and Psychological Responses in People with Terminal Cancer (1)†			●				
13	Crisis Intervention and Post-Incident Response	Information Sharing on Suicide Attempt Cases in the Emergency Department (1)			●				
		Information Sharing in Emergency Meetings (1)				●			
		Information Sharing During Crisis Intervention (1)			●				
		Methods for Providing Information to Bereaved Families and the Community (1)				●			
14	On-Site Response Procedures Upon Discovering a Suicide	On-Site Response Procedures Upon Discovery of Suicide (15)				●		•	
15	Communication and Reporting Procedures Upon Discovering a Suicide	Contact and Reporting Procedures Upon Discovery of Suicide (14)				●		•	
16	Post-Incident Procedures for Psychological Care for Bereaved Families and Staff	Psychological Support Procedures for Staff After an Incident (7)				●			•
		Psychological Support Procedures for Bereaved Families After an Incident (1)				●			
17	Post-Incident Debriefing and Staff Care System	Procedures for Conducting Emergency Meetings (3)				●			
		System for Providing Post-Incident Staff Support (2)				●			
18	Interdisciplinary Collaboration within the Hospital and with External Organizations	Multidisciplinary In-Hospital and External Organizational Collaboration (3)					●		•
		In-Hospital Counseling System (4)†					●		
		Importance of Suicide Prevention in Medical Institutions (1)					●		
		Post-Incident Support for Staff Across Disciplines (1)					●		
19	System Development (Management, Consultation, Support, Reporting, Education, etc.)	In-Hospital Systems for Suicide Prevention: Counseling, Education, and Support (1)					●		
		Post-Incident In-Hospital Management System (1)					●		
		Clarification of Departmental Responsibilities, Reporting Systems, and Education Structures (1)					●		
		System for Ongoing Post-Incident Support (1)					●		
20	Consultation Services for Suicide Prevention	Mental Health Consultation Services in Kyoto City (1)					●		
		Legal Support for Bereaved Families by the Suicide Survivor Support Center (1)					●		
		Legal and Administrative Consultation Services by Bar Associations (1)					●		

“Common” (C) refers to the six topics frequently described across hospitals, while “Hospital-Specific” (S) indicates the six topics reflecting the unique characteristics of suicide prevention efforts at specific hospitals; these are labelled as “C” and “S,” respectively, in the Type column of the table for brevity.

† next to a subgroup item indicates that the item includes at least one paragraph containing the word “cancer.”

The numbers in parentheses next to the subgroup items indicate the number of paragraphs included in each corresponding subgroup.

The term “cancer” appeared in 22 of the 153 paragraphs (14%), indicating that the content focused on people with cancer (**Table 2**). Within the Overview category, the content explained cancer-specific risk factors, as well as the epidemiology of suicide risk among people with cancer and the necessity of implementing suicide prevention measures in DCCHs. In the Prevention category, examples included the use of screening tools, such as a Questionnaire on Ease of Daily Living, and descriptions of suicide risk factors among people with cancer. In the Intervention category, the content addressed psychological responses such as grief experienced by people with terminal cancer. In the Systems category, descriptions focused on internal coordination systems, including the role of the cancer counselling and support center.

### 3.4 Topic types: common and hospital-specific

**Figure 1:**
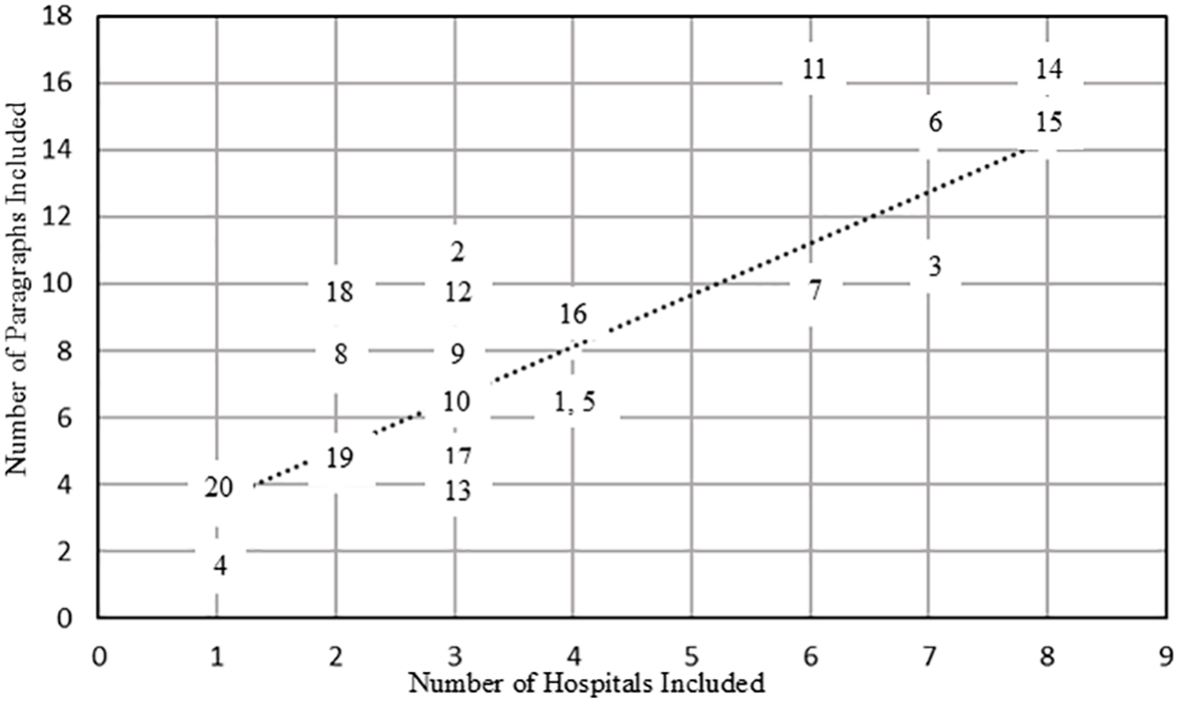
Number of paragraphs and hospitals per topic. The straight line in the figure represents the regression line derived from a simple linear regression analysis of the relationship between the number of hospitals whose manuals or workflows included each topic and the number of paragraphs in which the topic appeared (p < 0.01). The regression equation is as follows: Number of paragraphs = 1.980 + 1.543 × (Number of hospitals including the topic).

**Figure 1** displays the number of paragraphs associated with each topic in relation to the number of hospitals whose manuals or workflows included that topic. Six topics (3, 6, 7, 11, 14, and 15) were identified in six or more hospitals, representing content commonly shared across institutions. These topics encompass suicide prevention measures spanning the full continuum of prevention, intervention, and postvention, and were categorized as “Common” in the “Type” column of **Table 2**.

In contrast, six topics (2, 8, 9, 12, 16, and 18) appeared in the manuals or workflows of only a small number of hospitals but were located above the regression line. This indicates that although these topics were not widely addressed across institutions, they were discussed in greater detail by the hospitals that included them. Accordingly, these topics were classified as “Hospital-specific” in the “Type” column of **Table 2**. Further details on these hospital-specific topics, including their distribution by hospital, are provided in **Supplementary Figure 3**.

As a supplementary analysis, the topics derived from the primary analysis were externally applied to the paragraphs from Hospital K, which had been excluded from model training due to concerns regarding institutional non-independence. Topic-occurrence probabilities were estimated for each paragraph and aggregated in the same manner as in the primary analysis. When Hospital K was included in the regression framework using these externally applied topic probabilities, the positive linear relationship between the number of hospitals including a topic and the total number of associated paragraphs was preserved, although the absolute paragraph counts and the relative positions of several topics with respect to the regression line changed slightly. The results of this supplementary analysis are presented in **Supplementary Figure 4**.

### 3.5 Recommended components of the suicide prevention manuals or workflows for people with cancer

The 12 topics extracted in this study, comprising six commonly observed topics and six hospital-specific characteristics, were compared with the nine components of suicide prevention outlined in the general section of the NCC Guidelines (15), using these components as the reference standard for comparison. A detailed comparison, including the rationales for inclusion and exclusion, is provided in Supplementary **Table 1**. Based on these findings, we determined the recommended components for inclusion in suicide prevention manuals and workflows for individuals with cancer (**Table 3**).

**Table 3:** Recommended components for suicide prevention manuals and workflows for people with cancer.

Topic category	Topic type	Item	Description
Overview	Specific	Overview of Suicide Prevention Measures	Prevention, intervention, and postvention strategies for suicide Practical examples of suicide prevention: counseling, education, support, and systems
Prevention	Common	Types of Suicide Hotspots and Examples of Countermeasures	Examples of hotspot types and preventive measures
	Common	Risk Factors for Suicide and Assessment Methods	Risk factors for suicide Suicide risk assessment checklist (Source: Journal of Patient Safety Promotion, Issue 17)
	Specific	Screening Methods for Emotional Distress	Regular screening implementation (e.g., the Questionnaire on Ease of Daily Living)
Intervention	Specific	Examples of Responses to Suicide-Related Behaviors	Methods for responding to patients with suicidal ideation
	Common	Procedures and Collaboration System for Managing Patients with Suicidal Ideation	Procedures and coordination for providing care for patients with suicidal ideation
	Common	Examples of Communication Methods for Patients with Suicidal Ideation	Communication methods during crisis intervention
Postvention	Common	On-Site Response Procedures Upon Discovering a Suicide	On-site response procedures upon discovery of suicide
	Common	Communication and Reporting Procedures Upon Discovering a Suicide	Contact and reporting procedures upon discovery of suicide
	Common	Examples of Communication Methods for Bereaved Families and Other Patients	Communication with other patients and bereaved families after a suicide
	Specific	Psychological Reactions, Including Grief, and Related Support	Psychological reactions among bereaved families and corresponding support
	Specific	Post-Incident Procedures for Psychological Care for Bereaved Families and Staff	Psychological support procedures for staff and bereaved families after an incident
System	Specific	Interdisciplinary Collaboration within the Hospital and with External Organizations	In-hospital counseling system Multidisciplinary in-hospital and external organizational collaboration

“Common” and “Specific” refer to topics frequently observed across hospitals and those that reflect the unique characteristics of suicide prevention efforts at specific hospitals, respectively.

“Communication with Patients with Suicidal Ideation” and “Communication with Bereaved Families and Other Patients” are derived from the common topic “Communication Methods with Patients, Bereaved Families, and Other Patients” and are presented separately under intervention and postvention, respectively.

## 4 Discussion

This study applied LDA, a topic modeling method, to analyze the content of suicide prevention manuals and workflows for people with cancer from DCCHs. Twenty topics were extracted, encompassing commonly observed topics across multiple hospitals and hospital-specific topics described in greater detail by individual hospitals. Based on these findings, we identified recommended components for inclusion in suicide prevention manuals and workflows for individuals with cancer.

The common topics largely reflected the prevention–intervention–postvention framework, which has been emphasized in previous studies and major guidelines as essential components of suicide prevention (15, 20–22). Among the common topics, approximately 32% of the subgroups aligned with prevention, 29% with intervention, and 39% with postvention. Prevention-related subgroups were prominently represented by content such as “Examples of Hotspot Types and Preventive Measures”, indicating that many hospitals place emphasis on identifying high-risk environments and implementing preventive strategies. This focus aligns with evidence that restricting access to lethal means is an effective suicide prevention strategy (23). Intervention-related subgroups accounted for 29% of the common topics and were most prominently represented by “Communication Methods During Crisis Intervention”. This emphasis reflects the importance of multidisciplinary, team-based approaches, which have been shown to be more effective than usual care in improving depressive symptoms (24). Postvention-related subgroups constituted the largest proportion (39%) of the common topics and were particularly characterized by “On-Site Response Procedures Upon Discovery of Suicide”. This finding suggests that many hospitals place substantial emphasis on clear procedural guidance following a suicide, reflecting the practical and organizational demands of postvention in clinical settings.Conversely, the defining characteristics of hospital-specific topics reflected the distinct priorities and resources of each institution, demonstrating that notable differences emerged across hospitals. For example, in hospitals emphasizing collaboration with external organizations, manuals included specific accounts of coordination with community-based services such as financial support or employment support facilities. While only one example is presented here for clarity, further examples of hospital-specific topics are available in the **Supplementary Material**.

Considering the commonalities and differences observed across hospitals, researchers with expertise in suicide among people with cancer identified a set of recommended components for suicide prevention manuals and workflows for people with cancer. Because circumstances vary depending on the geographic location and scale of each hospital, institutions should use these components as guidance and incorporate the topics most relevant to their own context. These recommended components should guide the development of manuals and workflows and be integrated into the broader framework of cancer care delivery. Integrating suicide prevention efforts into the continuum of cancer care—from diagnosis and treatment to palliative care and survivorship—may help reduce the stigma associated with mental health and foster more comprehensive patient-centered support.

Importantly, although the components proposed in this study were derived from analyses of suicide prevention manuals and workflows in Japanese hospitals, the overarching framework of prevention, intervention, and postvention corresponds to core elements that have been consistently identified as essential to suicide prevention in prior international and theoretical literature (20, 21). Previous sociological research has underscored that risk factors and preventive strategies for suicide vary substantially across institutional and cultural contexts (25, 26), and a recent global meta-analysis of suicide in people with cancer demonstrated considerable variation in prevalence and risk across countries (27). While the specific content and operationalization of suicide prevention measures are inevitably shaped by local institutional, cultural, and healthcare system factors, the underlying framework appears to be broadly shared. Therefore, conducting international comparative analyses of suicide prevention manuals and workflows may help elucidate how this common framework is adapted across settings and identify both universally relevant and context-specific elements of suicide prevention. Future research should expand the sample size to include a broader range of institutions and examine the cross-cultural applicability and practical application effects.

This study had some limitations. First, as LDA analyzes texts based solely on word occurrence frequency, paragraphs containing similar keywords tend to be categorized under the same topic. For example, those frequently containing the word “communication” included communication during a crisis intervention (intervention) and communication with bereaved families (postvention), which were sometimes grouped within the same topic. Therefore, a human review was required to confirm and appropriately classify the topics. Second, the length of the manuals and workflows varied substantially across hospitals, ranging from a single-page workflow to a 28-page document. Such variation may have influenced which topics emerged through topic modeling and whether they were identified as common or hospital-specific. Third, when Hospital K—previously excluded from the determination of the regression line used to classify topics as common or hospital-specific—was included, the relative positions of several topics with respect to the regression line changed slightly. Therefore, further refinement and validation of the method used to classify topics as common or hospital-specific are warranted in future studies. Fourth, this study focused solely on analyzing the content described in the manuals and workflows without considering their practical implementation. Manuals are typically developed for specific contexts, and their applications may differ across institutions. Thus, future studies should examine how the recommended components of suicide prevention manuals are applied in clinical settings.

In conclusion, commonly shared topics should be prioritized for inclusion in suicide prevention manuals and workflows for individuals with cancer. Conversely, hospital-specific topics may serve as useful references tailored to the distinct characteristics of each hospital. Factors such as hospital size, geographic location, and clinical practice should be considered when determining the content of these manuals and workflows.

## Data Availability

The datasets presented in this article are not readily available because due to institutional confidentiality agreements with the participating Designated Cancer Care Hospitals. However, data may be available from the corresponding author on reasonable request and with permission from the hospitals. Requests to access the datasets should be directed to Maiko Fujimori, mfujimor@ncc.go.jp.
