# Supplementary material for: Suicide prevention in Japanese cancer care hospitals: a topic modeling analysis of manuals and workflows
Source: Front Psychiatry. 2026 Jan 21;17:1743690. doi: 10.3389/fpsyt.2026.1743690 (PMC12869307; doi:10.3389/fpsyt.2026.1743690)
Supplement: Supplementary file 1 [file DataSheet1.pdf]

Supplementary Material

Supplementary Table 1. Comparison between the nine recommended components from the 2019 edition of the Guidelines for Suicide Prevention in Cancer Care and the topics extracted through topic modeling

			Topics Identified in This Study	
			Common	Specific
The 2019 Edition of the Guidelines for Suicide Prevention in Cancer Care	Prevention	Enhancement of Consultation and Support Systems		18. System
		Education and Training Systems for Healthcare Professionals		2. Overview
		Countermeasures for Suicide Hotspots	3. Prevention	
		Dissemination of Knowledge		
	Intervention	Interventions for High-Risk Populations	6. Prevention	
		Screening and Risk Assessment	7. Intervention	8. Prevention
		Communication	11. Prevention, Intervention	9. Prevention, Intervention
		Multidisciplinary Case Management	7. Intervention	18. System
	Postvention	Support for Bereaved Families and Healthcare Professionals	11. Postvention	8, 9, 12, 16. Postvention
N/A			14, 15. Postvention	

Notes:

- In the topics extracted in this study, “Common” refers to the six topics frequently described across hospitals, and “Specific” refers to the six topics reflecting the unique characteristics of suicide prevention efforts at specific hospitals.
- The numbers in the table correspond to the topic numbers listed in Table 2.
- “Prevention,” “Intervention,” and “Postvention” correspond to primary, secondary, and tertiary suicide prevention strategies, respectively, and represent the five overarching categories, including Overview and System.
- Some topics span multiple components recommended in the guidelines and, therefore, appear in

multiple categories.

Additional Explanations:

Supplemental Table 1 compares the nine recommended components from the 2019 Guidelines with the topics extracted in this study. The dissemination of knowledge as part of preemptive measures is described in the National Cancer Center (NCC) Guidelines as follows:

> *"It is important to promote understanding of mental illness, enhance skills for assessing suicide risk, and disseminate knowledge about suicide prevention within communities through the training and continuing education of healthcare professionals. These are also considered essential components of suicide prevention in cancer care."*

This item is positioned as a broader suicide prevention strategy that extends beyond hospital-level initiatives; therefore, it was not included among the topics identified in this study and was excluded from the list of recommended items.

In contrast, Topic 14: "On-Site Response Procedures Upon Discovering a Suicide" and Topic 15: "Communication and Reporting Procedures Upon Discovering a Suicide" were not included among the recommended components of the NCC Guidelines. However, as noted in the characteristics of the common topics, the Japan Council for Quality Health Care designates suicide as a serious medical incident. Therefore, these topics—detailing institutional responses following the occurrence of suicide—are considered important components of postvention within hospital settings.

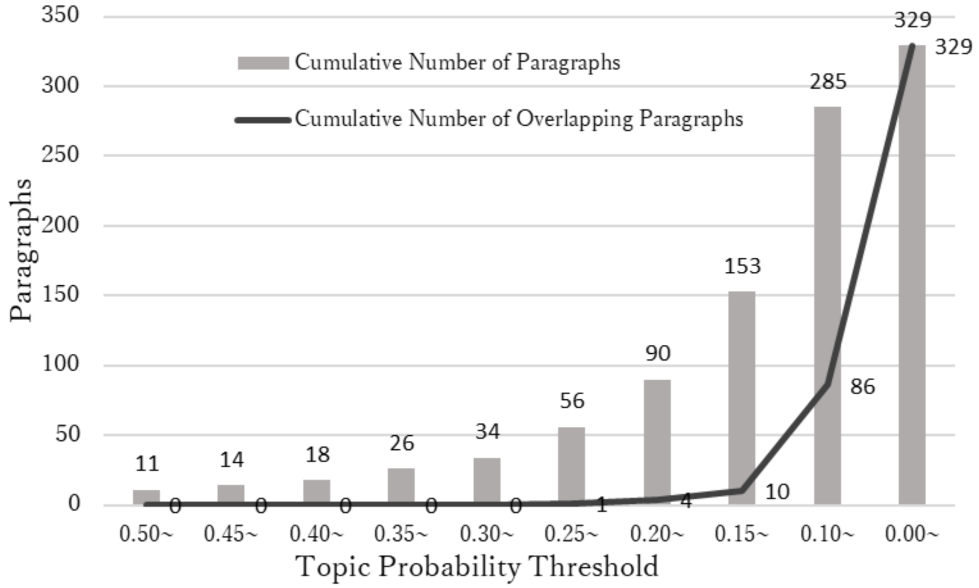

Supplemental Figure 1. Cumulative Number of Paragraphs by Topic Probability Threshold

Notes:

- The grey bars indicate the number of paragraphs in which the topic probability exceeded the threshold for each topic, while the black line indicates the number of paragraphs in which the same paragraph exceeded the threshold for multiple topics.

For example, the data point at 0.15 on the x-axis indicates that 153 paragraphs had a topic probability of 0.15 or higher. Among these, 10 paragraphs had two or more topics with probabilities exceeding 0.15.

Additional Explanations:

Supplemental Figure 1 illustrates how the number of paragraphs varies depending on the probability threshold applied. For example, when the threshold was set at 0.15, 153 paragraphs contained at least one topic probability at or above this value, while 10 paragraphs contained multiple topics exceeding the threshold. By contrast, at a threshold of 0.10, 285 paragraphs met the criterion, and 86 paragraphs contained multiple topics above the threshold. These results indicate that a higher threshold reduces topic overlaps and enhances interpretability but limits the number of eligible paragraphs. Conversely, a lower threshold increases coverage but reduces interpretability because of topic co-occurrence.

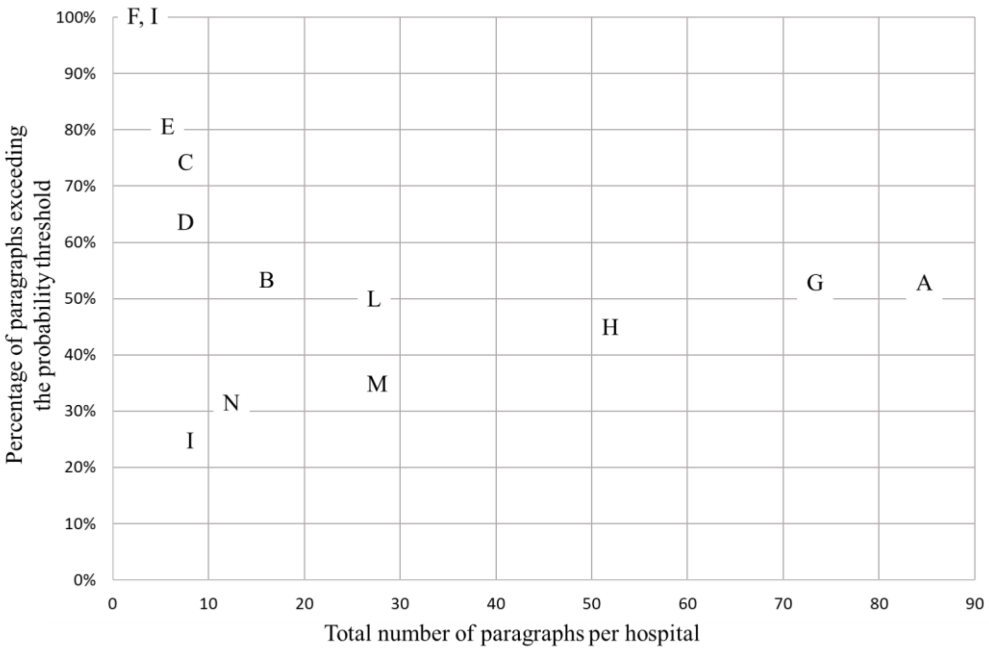

Supplemental Figure 2. Relationship between total number of paragraphs per hospital and the proportion of paragraphs exceeding the topic probability threshold

Additional Explanations:

This figure plots the proportion of paragraphs exceeding the topic-occurrence probability threshold (0.15) against the total number of paragraphs per hospital. The correlation coefficient between the total number of paragraphs and this proportion was −0.37, indicating a negative association. The distribution indicates variability across hospitals but does not suggest systematic underrepresentation of hospitals with smaller manuals.

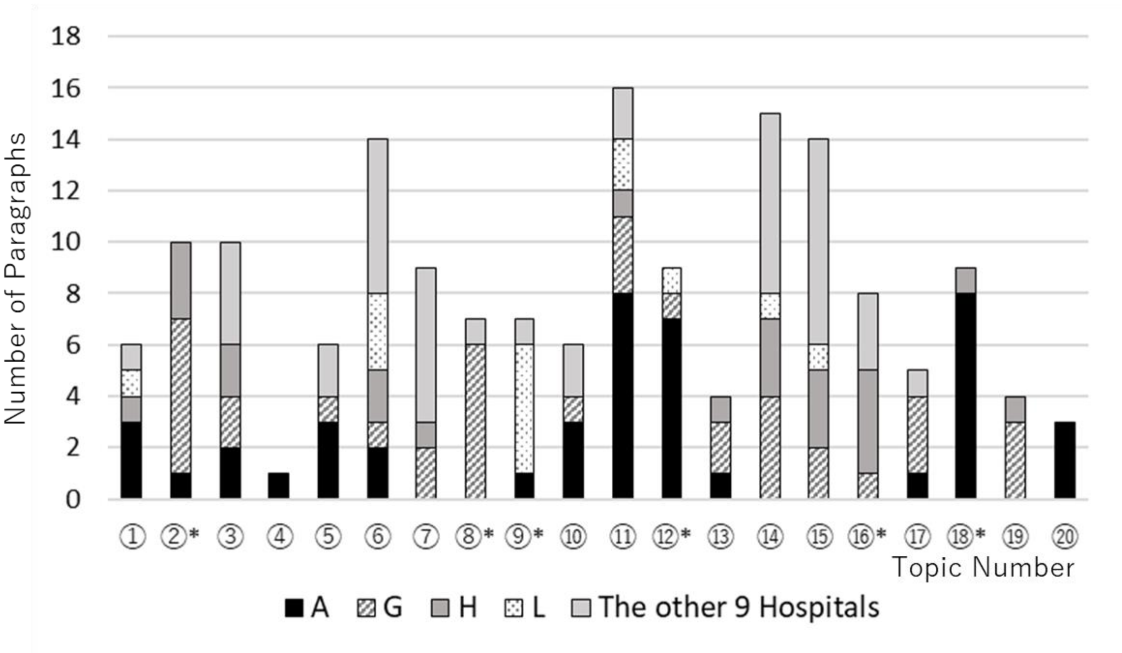

Supplemental Figure 3. Number of paragraphs per topic by hospital

Notes:

- Topic numbers marked with an asterisk (*) indicate topics reflecting hospital-specific characteristics.
- Hospitals A, G, H, and L are distinguished by different shading patterns from the other nine hospitals.

Additional Explanations:

Supplemental Figure 3 presents the distribution of each topic across hospitals. The asterisked topic numbers correspond to the six hospital-specific topics identified in this study. In each case, a single hospital accounted for more than half of the total paragraphs associated with that topic, indicating that these topics reflect distinctive suicide prevention practices specific to those hospitals.

- Hospital G included Topic 2 (Overview of Suicide Prevention Measures) and Topic 8 (Screening Methods for Emotional Distress).
- Hospital L included Topic 9 (Examples of Responses to Suicide-Related Behaviors).
- Hospital A included Topic 12 (Psychological Reactions, Including Grief, and Related Support) and Topic 18 (Interdisciplinary Collaboration within the Hospital and with External Organizations).
- Hospital H included Topic 16 (Post-Incident Procedures for Psychological Care for Bereaved Families and Staff).

Among these hospitals, Hospital L was a general hospital, whereas Hospitals A, H, and G were university hospitals.

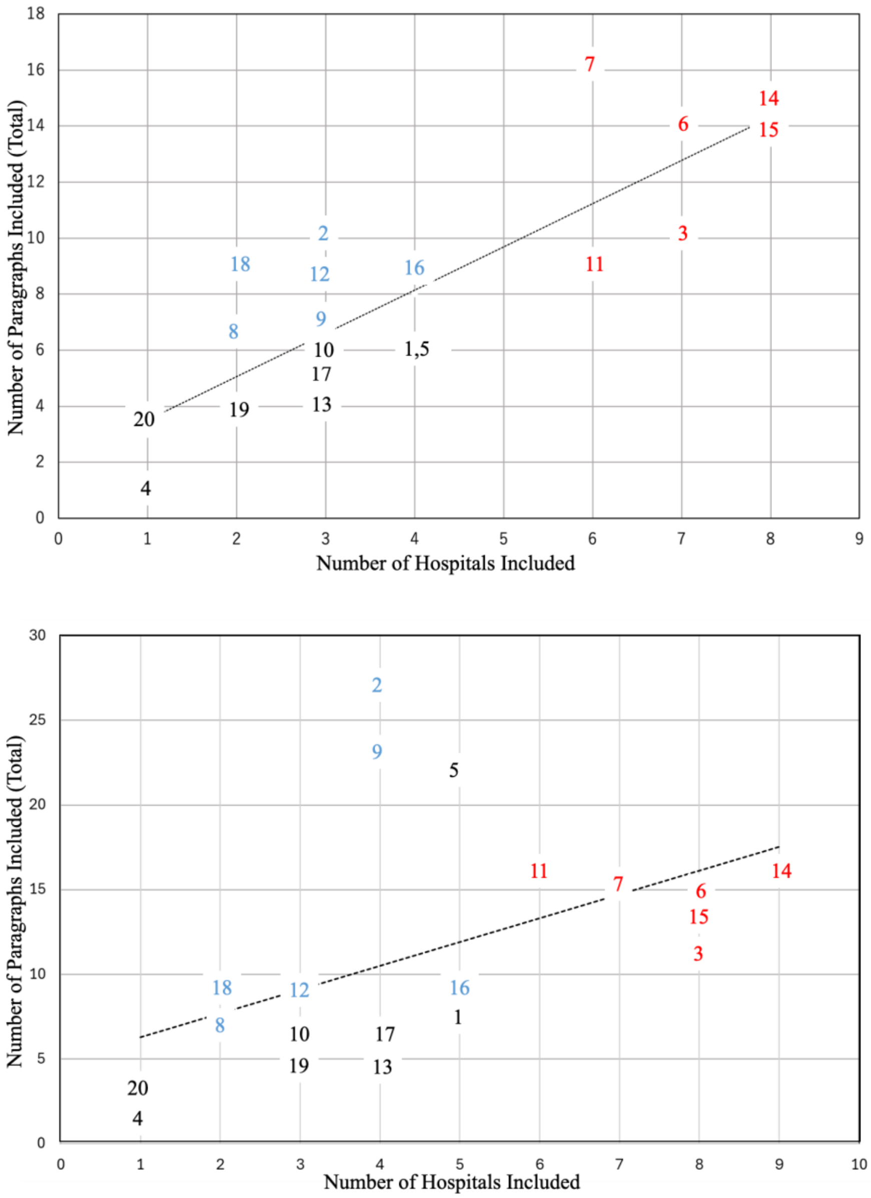

Supplemental Figure 4. Relationship Between the Number of Hospitals Including Each Topic and the Number of Paragraphs Assigned to the Topic

Notes:

- The upper panel shows the results of the primary analysis and is identical to Figure 1 in the main text. The lower panel extends the analysis by applying the latent Dirichlet allocation (LDA) model trained in the primary analysis (excluding Hospital K) to the paragraphs from Hospital K. Topic–paragraph occurrence probabilities for Hospital K were inferred using the trained model and aggregated with those from the primary analysis.
- The straight line in the figure represents the regression line derived from a simple linear regression analysis of the relationship between the number of hospitals whose manuals or

workflows included each topic and the number of paragraphs in which the topic appeared in the primary analysis.

- The regression equation (the lower panel) is as follows:
  Number of paragraphs = 4.882 + 1.406 × (Number of hospitals including the topic); $p < 0.05$.
- Topics shown in red indicate common topics, whereas topics shown in blue indicate hospital-specific topics.

Additional Explanations:

This supplementary analysis examines how paragraphs from a hospital not included in model training are probabilistically assigned to existing topics. By applying the trained LDA model without retraining, the figure illustrates how the inclusion of Hospital K affects the distribution of topics relative to the regression line derived from the primary analysis. Although the inclusion of Hospital K altered the absolute paragraph counts for some topics and led to slight shifts in the relative positions of several topics with respect to the regression line, the overall regression structure and interpretation remained unchanged.

Supplementary Information

This supplementary Information provides additional examples of hospital-specific topics, complementing the single example presented in the main text. These examples illustrate how institutional priorities and available resources shaped the manuals.

- **Overview of Suicide Prevention Measures**: Explains the objectives and significance of primary, secondary, and tertiary suicide prevention.
- **Screening Methods for Emotional Distress**: Describes procedures for sharing patients' mental health information—derived from the *Questionnaire on Ease of Daily Life*, a brief distress screening tool for cancer inpatients developed through the Ministry of Health, Labour and Welfare's regional palliative care project—among staff members, and emphasizes the importance of such information sharing in clinical practice.
- **Examples of Responses to Suicide-Related Behaviors**: Highlights the importance of exploring the underlying factors contributing to a patient's suicidal ideation when providing care.
- **Psychological Reactions, Including Grief, and Related Support**: Outlines the psychological reactions of people bereaved by suicide and the corresponding support systems, with attention to changes in grief over time.
- **Post-Incident Procedures for Psychological Care for Bereaved Families and Staff**: Underscores the importance of psychological care for staff alongside bereavement care for families, and specifies the procedures involved.
